# Modeling and Experimental Analysis of Shear-Slitting of AA6111-T4 Aluminum Alloy Sheet

**DOI:** 10.3390/ma13143175

**Published:** 2020-07-16

**Authors:** Łukasz Bohdal, Leon Kukiełka, Stanisław Legutko, Radosław Patyk, Andrii M. Radchenko

**Affiliations:** 1 Department of Mechanical Engineering, Koszalin University of Technology, Racławicka 15-17 Street, 75-620 Koszalin, Poland; leon.kukielka@tu.koszalin.pl (L.K.); radoslaw.patyk@tu.koszalin.pl (R.P.); 2 Faculty of Mechanical Engineering, Poznan University of Technology, 3 Piotrowo Street, 60-965 Poznan, Poland; stanislaw.legutko@put.poznan.pl; 3 Department of Ship Electroenergetic Systems, National University of Shipbuilding named after admiral Makarov, Heroiv Stalinhradu Ave, 9, 54025 Mykolaiv, Ukraine; nirad50@gmail.com

**Keywords:** aluminum alloy, shear-slitting, sheared edge, FEM modeling, SPH modeling, cutting condition, optimization

## Abstract

This work presents experimental studies with numerical modeling, aiming at the development of guidelines for shaping aluminum alloy AA6111-T4, *t* = 1.5 mm thick, with the use of a shear-slitting operation. During the experimental tests, parametric analyses were conducted for the selected material thickness. For the purposes of the material deformation’s analysis, a vision system based on the digital image correlation (DiC) method was used. Numerical models were developed with the use of finite element analysis (FEA) and the mesh-free method: smoothed particle hydrodynamics (SPH), which were used to analyze the residual stress and strain in the cutting zone at different process conditions. The results indicate a significant effect of the horizontal clearance between knives on the width of the deformation zone on sheet cut edge. Together with the clearance value increase, the deformation zone increases. The highest burrs on the cut edge were obtained, when the slitting speed was set to *v* = 17 m/min, and clearance to *h_c_* = 6%*t*. A strong influence was observed of the horizontal clearance value at high slitting speeds on burr unshapeliness. The most favorable conditions were obtained for *v* = 32 m/min, *h_c_* = 0.062 mm, and rake angle of upper knife for *α* = 30°. For this configuration, a smooth sheared edge with minimal burr height was obtained.

## 1. Introduction

The dynamic development of technology observed in recent years, is closely related to the search for such a manufacturing processes, that will ensure the required final product’ high quality, with minimum number of machining operations [1,2,3,4,5]. The mechanical cutting process is one of the most common methods of producing elements, and shaping parts in various industrial branches. High process efficiency allows the provision of a large number of parts within a relatively short production time. The development of cutting technology in recent years necessitates the improvement of analysis methods applied for this process. For a long time, difficulties associated with the strongly non-linear nature of such a process, did not allow to reach precise and universal methods of its analysis. In recent years, there has been an extremely rapid development in the field of continuous media theory, plasticity theory, and numerical methods of mechanics taking place, with the progress of computational systems and specialized software. This creates conditions in which the analysis of complex problems of plastic forming has become possible [1,2,3,4,5,6,7].

In the case of shear-slitting, the tools make a rotary movement. The rotation of each knife is influenced by the applied torque [8]. Quality of high cut edge is required for final workpiece. In the shear-slitting process, the mechanism of the material’s separation is often very hard to control. Difficulties arise from the necessity of precise settings of technological process parameters during the production cycle [8,9,10]. A large number of these parameters require specialized knowledge of their impact on the process as well as the mutual correlation between them. Sticking material to the surface of cutting tools, sheet edge wave, sheet twisting and bowing, formation of burrs, slivers, and debris on the cut edge constitute the main difficulties while forming light metal alloys on production lines [11,12,13]. The selection of slitting parameters is done by trial and error, which increases the process’ duration and the amount of waste. In case of the cut edge’s defects, smoothing and deburring operations are used, which increases the costs of production. High sheared edge burrs can scratch the sheet surface during storage, and it can lead to polyurethane clamping roll’s damage during the process [10,14].

Some studies have been conducted to understand the shearing mechanisms of construction steels. The effect of shearing process conditions on sheet deformation, stress variables, fracture mechanisms and sheet defect problems based on guillotining, blanking and trimming processes was examined [15,16,17,18,19]. Analyses and experimental tests of the finite element method (FEM) were carried out aiming at the improvement of the cutting process. In [11], a sensitivity analysis of the main shearing process parameters on sliver formation mechanism was conducted. Influencing parameters were studied based on the AA6014 aluminum alloy, resulting in the obtaining of the optimized process parameter configurations. Le et al. [20], taking into account sheet metal prestrain, analyzed the influence of cutting clearance and tool cutting angle on sheared edge formability of aluminum. Nasheralahkami et al. [21], based on advanced high strength steel, studied the effect of clearance and tool wear on trimmed edge formation, and the impact of process parameters on burr defect was investigated. Golovashchenko and Wang [16,22] investigated tensile stretching of aluminum, edge cracking and burr formation problems of material, that had previously been trimmed. In [22], Golovashchenko presented the results of metal sheared edge stretchability and fracture mechanism during trimming. Hilditch and Hodgson [23,24], using FEM simulations and hardness contours of cut samples, analyzed the shearing behavior of an aluminum alloy sheet. According to the analysis of the state of knowledge, some modifications can be made to the shearing process, aiming at a reduction in defects at the cut surface. Golovashchenko [13] proposed supporting of the cut sheet during trimming for the AA6111-T4 aluminum alloy. The influence of the trimming clearance on the cut edge quality was investigated. According to author, supporting can reduce the burr height. Paper [25] presents physical and mathematical models in the case of steel bundle cutting. There was discussed the influence of cutting parameters on deformations, strains and stresses in bundles of cold-rolled steel sheets and mechanism of fracture process. Gąsiorek et al. [26] presented a numerical modeling and parametric study of the cutting process of sheet aluminum bundles on a guillotine. An FEM, smoothed particle hydrodynamics, and Lagrangian-Eulerian approach were coupled to simulate the physical phenomena during the cutting process.

Currently, the knowledge of aluminum alloys shear-slitting mechanisms is very limited. Some experimental methods, which are often expensive and hard to be calibrated, are presented in the literature. In the works [27,28,29], the influence of cutting process conditions on the sheared edge quality of aluminum car body panels was examined. The work [9] presents mathematical models, describing physical phenomena occurring at the time of cutting parts of motor vehicles. The models allow for calculation of cutting forces depending on the adopted tool geometry. In the work [10], the influence of the cutting knives geometry and cant angle was analyzed with respect to the plane of the sheet on the quality of the cut surface of the 1050-H18 aluminum alloy. It was proven that the value of the knife rake angle has a high impact on the height of the burrs on the cut surface. Depending on the cant angle, it is possible to increase the quality of the cut edge of the sheet’s cut part. Unfortunately, it is difficult to control the quality of the cut off part’s sheared edge. Hubert et al. [30] analyzed stress fields in the sheet metal cut edge due to the edge trimming process by two rotary knives and revealed during the cold rolling process. For the purposes of modelling the edge trimming phases, the FEA was used. In order to obtain realistic stress variables and crack profiles, special attention was drawn to sensitivity analysis and meshing procedures.

The objective of this article is to study the impact of the main shear-slitting technological process’ parameters on the cut surface formation mechanisms and final quality of sheared edge of AA6111-T4 aluminum alloy. The numerical investigations were conducted with the use of the finite element method, mesh-free method SPH, and hybrid approach FEM-SPH. The results were compared with experimental investigations with the use of the vision system combined with the DiC method and scanning electron microscopy (SEM). The graphical optimization was employed using MATLAB software. The optimal point of the process efficiency was determined following the cutting process’ optimization. The obtained results can be of great significance to the control of the material sheared edge’s properties on production lines.

## 2. Experiment Setup and Results

**Figure 1:**
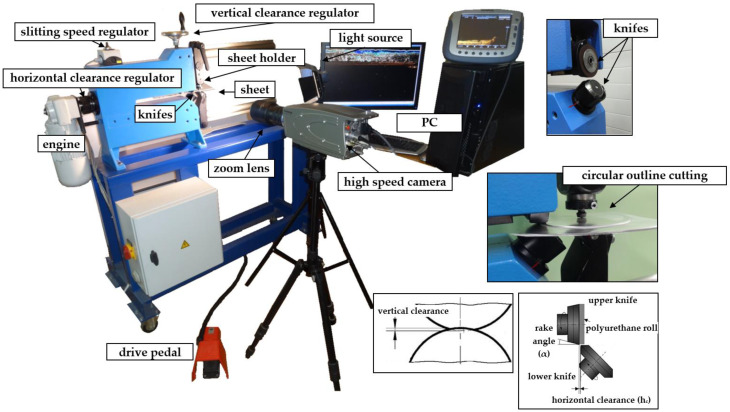
Experimental equipment.

The experimental research was conducted on the KSE 10/10 slitting machine (Figure 1) (Prinzing Maschinenbau, Lonsee, Germany) localized at the Koszalin University of Technology. The device consists of two rotary knives, an engine, a clamping roll, and special mounts with a sheet holder. The contact between the knives and sheet is considered to be a non-sliding one. The cutting process is realized by the rotation of knives and a polyurethane roll, which move the sheet in the horizontal direction. The horizontal clearance *h_c_*, defined as the distance between the upper and the lower knife cutting edges, is set by a clearance regulator with a scale. The slitting velocity is set by a knob with a scale. The scales are very accurate, which enables the precise setting of slitting parameters.

**Figure 2:**
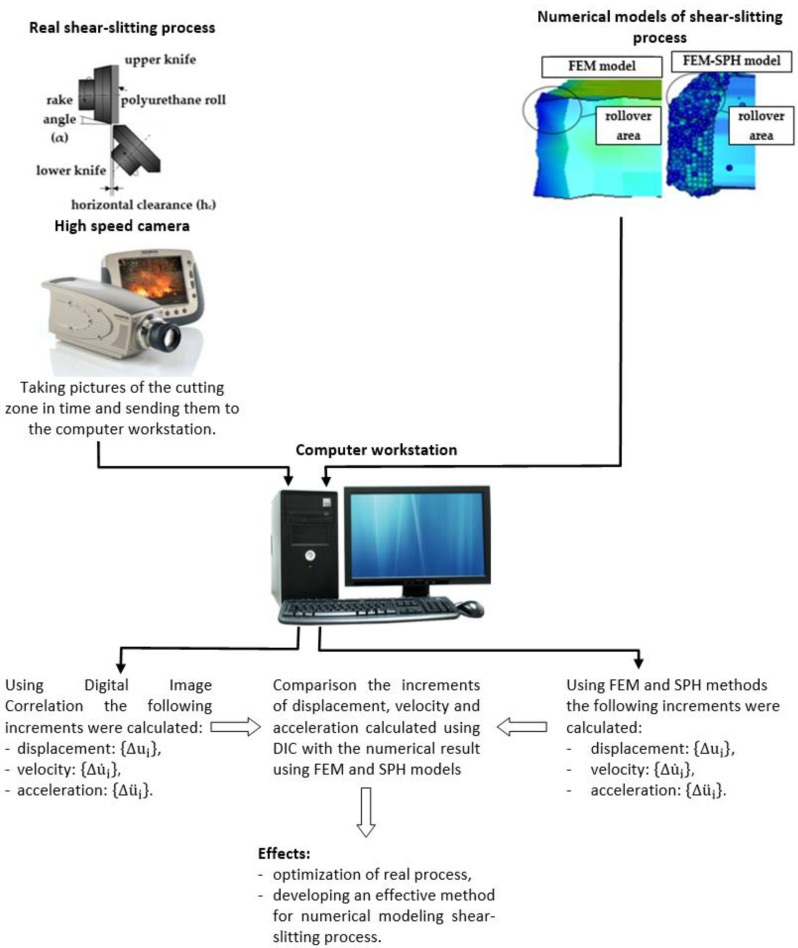
Research scheme.

An advanced vision-based method (digital image correlation) was used to observe material’s deformation and fracture mechanisms in the sheared zone (Figure 1 and Figure 2). A specially designed monitoring system (Koszalin University of Technology, Koszalin, Poland) was applied to record a sequence of images during the process. The images were captured at a resolution of 1280 × 1024 pixels, and at the speed 2000 fr/sec. High-speed camera *i*-SPEED TR (iX Cameras, Bradley House Locks Hill Rochford Essex, UK), i-SPEED Suite and the GOM Correlate software, were used for material displacement and strain measurement in shearing region. Specially chosen macro lenses and rings, combined with a light source, were used for investigations.

The mechanical and physical properties of AA6111-T4 aluminum alloy with thickness *t* = 1.5 mm, which are often employed for production of car body panels, doors, fenders and other smaller parts in automotive industry, are shown in Table 1 and Table 2. The values of the shear-slitting variables with the ranges of variation, are presented in Table 3. The following parameters, such as the slitting speed (*v*_2_), the horizontal clearance (*h_c_*), and the rake angle of the upper knife (*α*), are usually controllable on industrial lines. Experiments were conducted based on the classical experimental design method, with the use of five-level rotatable plan of experiment, that is presented in Table 4. The research was planned with the use of the E-Planner program (Sławomir Kukiełka, Leon Kukiełka, Koszalin University of Technology, Poland). The required number of experimental points amounted to 20. The tests were carried out for three replications for each plan level.

### 2.1. Mechanism of Slit Edges Generation

In this section, with the use of a monitoring system and a DiC method, we present sample results of experimental analyses. The proposed method makes it possible to track the material’s flow and a cracking path. The DiC technique is widely used in static and fatigue tests, it is a non-contact and non-interferometric optical method applied for measuring the structural elements’ surface deformation. References [31,32,33,34] present the implementation of the DiC method for the selected laboratory tests of building materials. This method has also been used to measure local plastic deformation in the material during uniaxial tension [35,36]. Some researchers measured plastic deformation and strain variables in the shearing processes with the use of DiC. Goijaerts et al. [37] measured the local strain values in the shearing zone during blanking process at low speed. Experiments were conducted with the use of 1 mm thick stainless steel sheets. Ghadbeigi et al. [38] analyzed the evolution of local deformations during thin high Si electrical steel sheets’ blanking. Hu et al. [39] analyzed the influence of trimming die clearance on the predicted tensile stretching ductility of aluminum alloy sheet. They used the DiC method to observe the maximum strain areas that were obtained near the fracture. Stretchability decreased with the increasing trimming clearances. In [40], the authors used DiC for the validation of FEM models constructed to simulate plastic deformation and ductile fracture of sheet metal during blanking. With the use of DiC, the distributions and developing trends of effective strain and damage were predicted.

**Figure 3:**
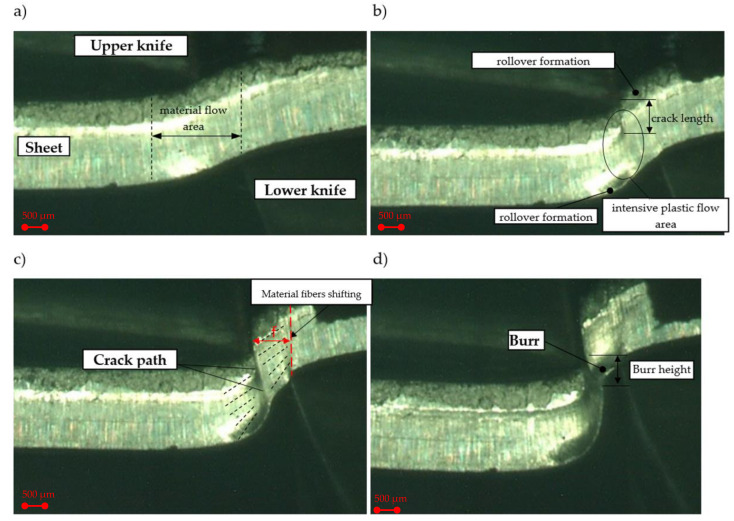
Shear-slitting process registered at the sheet cross section (h_c_ = 0.15 mm, α = 7°): (**a**) material plastic flow phase, (**b**) plastic flow with the beginning of the cracking phase, (**c**) burr formation in cracking phase, (**d**) final separation.

**Figure 4:**
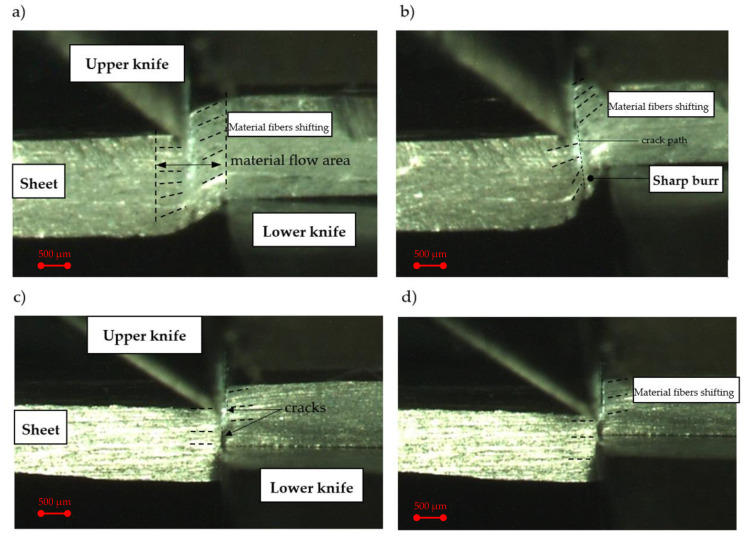
Shear-slitting process registered at the sheet cross section (α = 40°): (**a**) material plastic flow phase (h_c_ = 0.15 mm), (**b**) cracking phase (h_c_ = 0.15 mm), (**c**) plastic flow with cracking phase (h_c_ = 0.03 mm), (**d**) final separation (h_c_ = 0.03 mm).

A review of the literature also indicates that numerous practical problems arise due to the proper calibration of resolution of the DiC measurement, which depends on the surface pattern’s quality. At the beginning, a tested specimen need to be prepared applying a unique pattern of random speckles/dots on its surface. The speckle pattern can be the natural texture of the specimen surface [31,32,41]. In this work, we have conducted many tests to obtain reliable DiC conditions. Then, during the process, the observation of the characteristic zones’ formation on the cut surface and its defects formation was possible (Figure 3 and Figure 4). The results presented in this work show that it is possible to apply the DiC method to analyze the physical phenomena in the shearing zone during the shear-slitting process.

The images (Figure 3) show the different phases of the process in the selected experimental process’ conditions. At the initial stage of the process, it can be observed that the material’s flow area occurs not only in the cutting zone, but also out of it (Figure 3a). The material’s flow outside the cutting zone is caused by an inappropriate selection of rake angle, and horizontal clearance values. The presented parameters’ configuration, results in a rapid increase in the bending moment and the formation of a sheared edge’s rollover zone in the initial phases of the process. The rollover causes propagation of cracking only from the upper knife’s cutting edge, that runs outside the cutting area and is impossible to be controlled (Figure 3b,c). This causes a high curved burr (Figure 3d). A wide deformation zone and high material fibers’ shifting can also be observed. As a consequence, on the sheared edge accumulation of high deformations occurs. Li [12] analyzed the trimming mechanism of autobody sheet depending on the rake angle of the trim tool. Analyzing the results presented in the work [12] and our results, it can be concluded that a higher rake angle leads to a greater concentration of contact pressure and high plastic deformation around the cutting edge of the angled tool (Figure 4a).

Increasing the rake angle *α* at the maximum clearance *h_c_* = 0.15 mm reduces the material flow area and deformation-affected zone with material fibers’ shifting, but speeds up the material cracking phase (Figure 4a). The cut surface is more perpendicular to the sheet thickness, but is characterized by a sharp burr (Figure 4b). The bending moment and rollover area in this case are reduced. A reduction in the clearance to the minimum value *h_c_* = 0.03 mm resulted in a significant reduction in the deformation-affected zone and material fibers’ shifting (Figure 4c). Cracking, in this case, starts both from the upper and lower knife cutting edges, and runs in a straight line, perpendicular to the thickness of the sheet. In this case, rollover and burr are minimal (Figure 4d).

### 2.2. Quality of Sheared Edge

**Figure 5:**
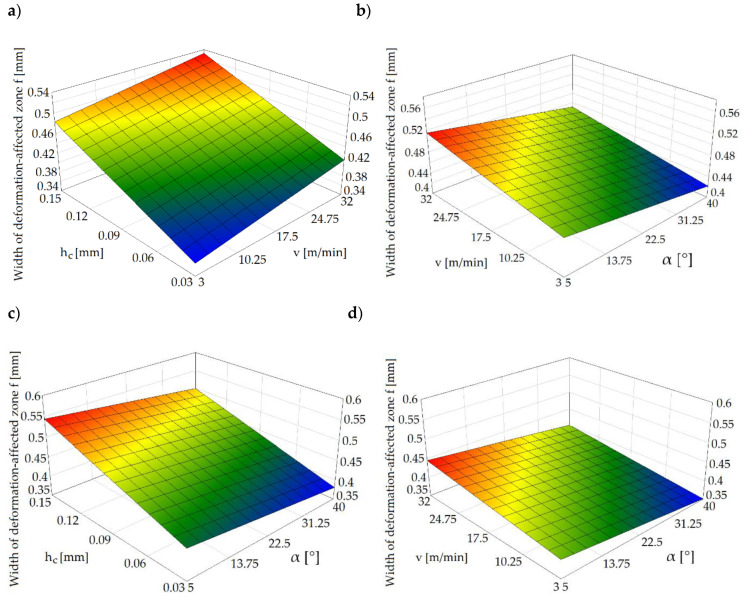
Graphs of the deformation-affected zone’s width for: (**a**) α = 30°, (**b**) h_c_ = 0.1 mm, (**c**) v = 20 m/min, (**d**) h_c_ = 0.03 mm.

The quality of sheared edge is analyzed in two steps. In the first step, depending on the given process parameters, the deformation-affected zone *f* near the cut surface is analyzed (Figure 3c). In the second step, their impact on the cut edge profile and burr height is determined. Figure 5a shows the effect of horizontal clearance and slitting speed on the width of the deformation zone.

The results indicate a significant impact of the horizontal clearance on the width of the deformation-affected zone. Along with the increase in the clearance value, the deformation zone increases, which is also presented in Figure 3c. The maximum width of the deformation-affected zone amounts approximately to 36% of the sheet thickness *t* (Figure 5a). A similar trend was observed in the work [42], in which the author analyzed the effect of trimming clearance on the width of the deformed zone of 5005 aluminum alloy cut on a guillotine. The increase in slitting speed caused a slight increase in the deformed zone’s width for small clearances (*h_c_* = 0.03–0.05 mm). The simultaneous increase in the slitting speed and rake angle value resulted in reduction in the deformed zone (Figure 5b). However, when using high slitting speeds (*v* > 24 m/min) and clearances (*h_c_* > 0.12 mm), it is unfavorable to use rake angle values from the range of *α* = 5°–20°. Selecting the rake angle values from the range of *α* = 30°–40°, results in less sensitivity of the deformation-affected zone’s width than clearance and slitting speed values (Figure 5c,d). However, for all rake angle values, an increase in clearance results in an increase in the width of the damage zone.

**Figure 6:**
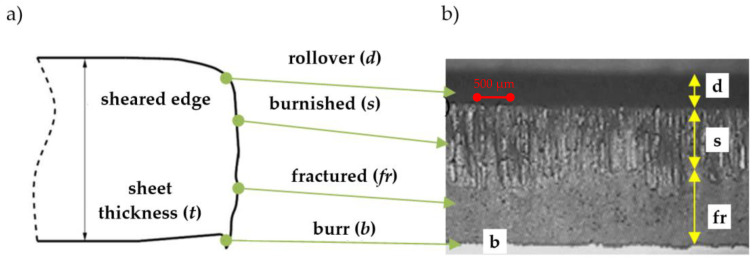
Typical sheared edge contour: (**a**) cross-sectional scheme, (**b**) the one obtained experimentally (front view).

Cutting of the sheet with the use of mechanical cutting, leads to a typical sheared edge profile in the workpiece, that can be separated into four zones (areas): Rollover, burnish, fracture and burr (Figure 6). The quality of the final product is determined based on the size of these zones. A high cut edge quality is correlated with the maximum burnished zone length, the minimum rollover, the fracture zones length, and the minimal burr height [43].

**Figure 7:**
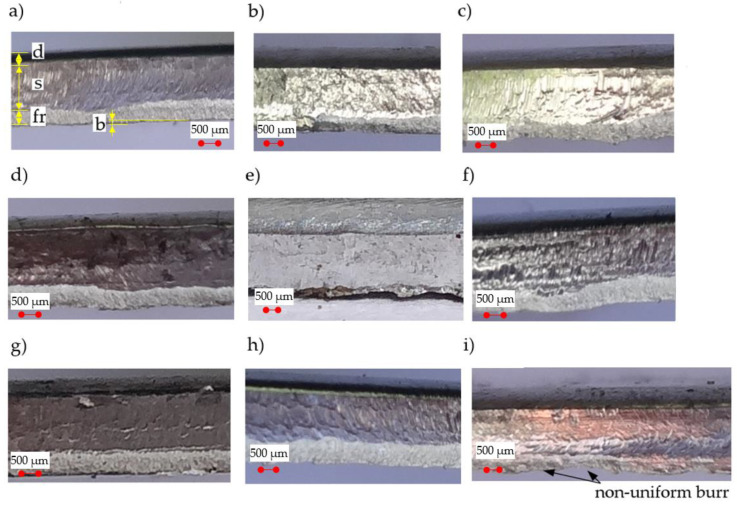
Characteristic features of the obtained sheared edges: (**a**) v = 7 m/min, h_c_ = 0.05 mm, (**b**) v = 27 m/min, h_c_ = 0.05 mm, (**c**) v = 7 m/min, h_c_ = 0.13 mm, (**d**) v = 27 m/min, h_c_ = 0.13 mm, (**e**) v = 3 m/min, h_c_ = 0.09 mm, (**f**) v = 32 m/min, h_c_ = 0.09 mm, (**g**) v = 17 m/min, h_c_ = 0.03 mm, (**h**) v = 17 m/min, h_c_ = 0.15 mm, (**i**) v = 17 m/min, h_c_ = 0.09 mm.

**Figure 8:**
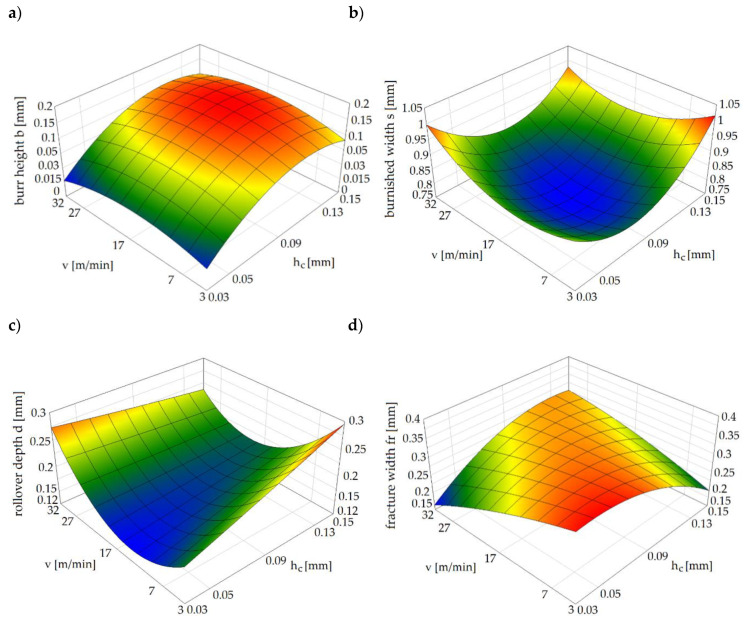
Influence of the selected process parameters on the: (**a**) burr, (**b**) burnish, (**c**) rollover, and (**d**) fracture zones.

We present the selected sheared edges of samples received for a constant rake angle *α* = 30° viewed by “Vision Engineering” optical microscopy in Figure 7. The values of the zones (Figure 6) were measured from the selected locations above the cut edge in the z-direction, averaged and presented in Figure 8.

Figure 8a shows the impact of the slitting velocity and clearance on the burr height. The general trend observed in the light alloys during the shearing process is for the burr height of the work piece, which increases together with the increase in cutting clearance [12,23,24]. Lu et al. [10], for the shear-slitting plastic materials, observed that together with the slitting speed increase, the burr height decreases. The results at lower speeds show a less sensitive effect in burr height for plastic materials. From our results it can be concluded that for all the analyzed slitting velocities, the increase in the clearance from *h_c_* = 0.03 mm to *h_c_* = 0.09 mm results in an increase in the burr height. The highest burrs are reached when the slitting speed is set to *v* = 17 m/min, and clearance is set to *h_c_* = 6%*t*. It is necessary to use deburring operation, because in this case the height of the burrs is non-uniform along the line of shearing (Figure 7i). According to the results presented in [10,12,44], burrs can become separated from the cut part and damages of edges visible in the form of transverse cracks will be formed. It could be a result of a significant gradient of clearances between tools along line of shearing. The formation of local burrs is an important problem occurring on the production lines, because they can tear off from the sheared surfaces increasing perpendicularity deviations. Because the burr formation is associated with the final rapture, much depends on the plasticity of the material. Higher ductility would lead to delayed rapture and formation of a burr higher than the one formed in a less ductile material [45]. So, it is very important to choose a proper value of a horizontal clearance and a rake angle of cutting tool. In guillotining processes, the use of small rake angle values and clearances for high ductility materials decreases the burr height and its unshapeliness, but increases the cutting forces.

In Figure 8b, we present a graph of burnished width. Results have shown a high impact of selected parameters on this zone and its surface structure. When the horizontal clearance is set to *h_c_* = 6%*t*, the burnished zone is highly reduced (Figure 8b) and the velocity effect is reinforced. A high burnished width (s = 66%t) is obtained when clearances of *h_c_* = 2%*t* and *h_c_* = 10%*t* are used. In these two cases, the effect of velocity on the burnished width is reduced. High velocity results in a course of plastic flow phase during shear slitting. This phase is less steady over a high range of velocities (*v* = 27–32 m/min) and characteristic peaks with the transition to sliding fracture can be observed.

Analyzing, guillotining, trimming and blanking processes [13,15,16,18] make it evident, that a bending moment occurs for any horizontal clearance. Contact stresses are distributed along a certain area of the cutting tools’ shearing edges and the cut material. As already indicated above, a bending moment has a strong influence on rollover formation on the sheared edge. Interestingly, at small slitting velocities, increasing the horizontal clearance increases the rollover, while at high velocities, increasing the clearance decreases the rollover (Figure 8c). As a result, the highest values of this zone are obtained when *v* = 3–7 m/min, or high velocities *v* = 32 m/min are used.

When the horizontal clearance is small, cracks are generated from the both tools, and they propagate along a straight line, while when the clearance is large, the cracks only propagate from the upper or lower tool side and open the mouth. It depends on the stress state at the shearing region, which is affected by clearance between cutting edges of the tools [46]. During the cracking phase, the initiated crack propagates through the rest of the material thickness by (initial) stable growth or by becoming unstable. During the unstable phase, there is a possibility of taking by the crack the form of multiple paths, which results in a secondary burnish zone. Our experimental studies show, that this zone does not occur for the analyzed process parameters. The material’s cracks proceed steadily and without multiple cracking paths. This research shows that the fractured area depends on both the horizontal clearance and slitting velocity (Figure 8d). The smallest fractured zone values are obtained by using high cutting velocities (*v* > 24 m/min) and small clearances (*h_c_* = 0.03–0.05 mm). For small cutting velocities, is possible to reduce the fractured zone with a clearance of *c* = 10%*t*.

## 3. FE and SPH Modeling

Shear slitting involves rotation of blades, and differs from the other shearing operations, such as punching and blanking. In this process, the material is simultaneously cut in two directions instead of one [47]. Three-dimensional analysis is required for complex process analysis. Some of the researches have studied the process using FEM. Wisselink and Hu’etink [44], in order to calculate the residual stresses and strains in a steady state of such a process, by using the Arbitrary Lagrangian Eulerian (ALE) formulation worked out a finite element model of slitting. A simple, uncoupled damage model was used to characterize the material’s behavior. Zhao et al. [48] worked out a FE model of galvanized sheets’ disc slitting process. This model was used for deformation, fracture, calculation of shearing force, and for material effective stress distribution during the process of cutting. Zhang [49] conducted a study of proper calculation and selection of energetic parameters for disc shears. Ding et al. [50], developed a two-dimensional finite element model of metal sheets’ shear-slitting. The shear failure criteria, combined with the element-deletion method, was implemented to the model. The effect of clearance on the burr height was analyzed. Ghozzi et al. [51] analyzed a double slitting process used to cut thick metal sheets. In the study, the influence of the main process parameters, such as the tool geometry and sheet thickness on cut edge formation and cutting force, was analyzed.

In this work, three-dimensional FE and SPH models of shear-slitting were developed in the FE software LS-Prepost package. The material formed in the cutting processes was subjected to very complex loading conditions. In order to analyze this process, it is necessary to use an advanced mathematical apparatus, including computer methods of mechanics. Thanks to this, it is possible to solve the problems with many variables, including nonlinearities. The shear-slitting process should be considered as a geometrically and physically nonlinear boundary—an initial problem, in which there are boundary conditions that are nonlinear, movable and changeable in time and space, and which are unknown in the areas of contact between the tool and the object. An updated Lagrange’s description was used to describe the non-linear phenomena in the shearing region on a typical incremental step time. The aim of incremental analysis is to determine the geometry of the cut material, and the state of its velocity increment, displacements, accelerations, stresses, strains, strain rates, etc., in the subsequent, discrete moments of time τ = 0, Δt, 2Δt,…, corresponding to a certain small increase in time. The increments of strains and stresses were described respectively together with an increment of a non-linear strain tensor of Green–Lagrange, and an increment of the second symmetric stress tensor of Pioli−Kirchhoff. As far as the non-linear process analysis is concerned, there are many significant problems appearing in the given incremental description. They concern the selection of appropriate coordinate systems, defining measures of deformation and stress, and their increments, as well as determination of the rules of their accumulation at each incremental step. A variational function was used for the purposes of a variational formulation of the object movement’s incremental equation. The mathematical model was supplemented with the uniqueness conditions [52]. The explicit integration method was used in the analysis. Variational methods are used in continuum mechanics to formulate the equations of motion [53]. Kukiełka [54,55,56,57,58,59] used variational method to formulate the equations of motion for a typical incremental step and for nonlinear problems in metal forming processes, because there is nothing to account for the incremental formulation. In [5,28,59,60], we present the detailed modeling algorithms and mathematical formulations applied to cutting processes. In this paper, this method has been used to model the shear slitting process.

We have developed an equation of motion for three-dimension body in the global Cartesians coordinate, by using the updated Lagrange incremental formulation. Assuming that a numerical solution has been obtained at discrete time points Δt, 2 · Δt,…, where Δt is the short step time, the solution for t + Δt is required. In this case, the incremental functional was formulated for increment displacement $\Delta F\left(\left\{\Delta {\ddot{u}}_{i}\right\},\;\left\{\Delta {\dot{u}}_{i}\right\},\left\{\Delta u_{i}\right\}\right)=\Delta F\left(\cdot \right)$, where $\left\{\Delta {\ddot{u}}_{i}\right\},\;\left\{\Delta {\dot{u}}_{i}\right\},\left\{\Delta u_{i}\right\}$ are the increment components of the acceleration, velocity, and displacement vectors, respectively. The variational equation of the object’s motion was obtained by using the stationarity conditions of the functional ΔF(·). Then, by applying the FEM discretization and incremental decompositions, and approximating the velocity $\dot{r}$ and acceleration $\ddot{r}$ vectors in terms of displacement **r** vector with the central difference method (DEM), and by using the following approximations:

(1) {r˙t}=12Δt({rt+Δt}−{rt−Δt}),⋯{r¨t}=12Δt({rt+Δt}−2·{rt}+{rt−Δt}),

the equation of motion of discrete object was obtained:

(2) [M˜]·{rt+Δt}={Q˜}

where $\left[\tilde{M}\right]$ is the effective mass matrix:

(3) $$\left[\tilde{M}\right]=a_{0}\left[M\right]+a_{1}\left[C\right]$$

as the sum of the mass [M] and the damping [C] matrixes of a discrete object and $\left\{\tilde{Q}\right\}$ effective load vector:

(4) {Q˜}={F}+{R}+a0[M]·(2·{rt}−{rt−Δt})+a1[C]{rt−Δt}

where {F} and {R} are the internal and external force vectors, respectively, with the integration constants: $a_{0}=\frac{1}{\Delta t^{2}}$ and $a_{1}=\frac{1}{2\Delta t}$.

From Equation (2) the desired displacement vector {rt+Δt} at the end of the step is obtained.

(5) {rt+Δt}={Q˜}·[M˜]−1

The procedure is repeated for each step in a given time interval $\left[t_{p};t_{k}\right]$.

The smoothed particle hydrodynamics method uses discretization, which does not require division of the analyzed body by means of a grid, as it is in FEM. The method uses kernel approximation. The detailed SPH modeling procedure is presented by us in [5]. In the first place, solving the numerical problems requires the discretization of the domain, for which the equations have been defined. In the next stage, the method for each point approximates each variable from the allowable space of the function and its derivatives. The approximation function is aimed at presenting partial differential equations in the form of a system of ordinary differential equations in a discrete form, with time variables. The SPH method is based on the smoothing function (describes the state parameters occurring in a particle), and on a set of discrete elements arranged in the studied space that the smoothing function is assigned to. In the SPH method, the maximum distance, at which the interaction occurs between the particles is called smoothing length, and the distance between particles is *d* (particle density). Proper discretization of the domain, as a result of which a set of particles is obtained, constitutes a very important step. For this purpose, several simulations were carried out for different discretization variants. From the point of view of reducing calculation time, a new hybrid approach was proposed. Tools-sheet contact areas, were described by SPH method with high density of particles. Non-contact areas were discretized with the use of FEM.

**Figure 9:**
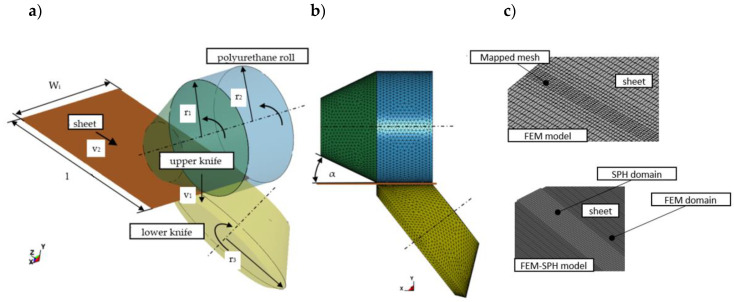
FEM and FEM + SPH simulation models of the shear-slitting process: (**a**) isometric view, (**b**) front view, (**c**) discretization of sheets.

**Figure 10:**
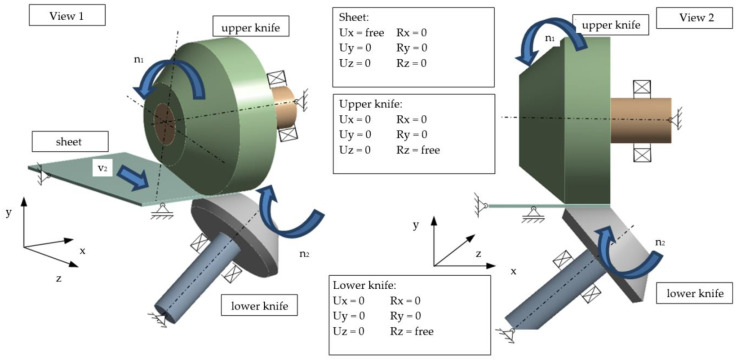
Boundary conditions for shear-slitting model.

According to the experimental configuration of the test stand, the FEM and FEM-SPH simulation models shown in Figure 9 and Figure 10 were created.

In the previous studies, we conducted analyses of blanking process by using the SPH method [5]. Studies showed that the results of the simulation are very close to the experiments in the material deformation’s characteristics and prediction of cutting forces. Simulations were carried out for spatial stress and strain states in the material. The developed application for the 3D modeling of the shear-slitting process allows for inclusion in the analysis of many technological parameters omitted in two-dimensional calculations, such as: length of the cutting line, value of the rake angle (α), knives radius values, method of fixing the sheet.

The process conditions for the numerical model are as follows: in the first phase of the process, vertical velocity (*v*_1_ = 200 mm/s) is applied so as to obtain the separation of the material in the cross-section. As a result of knives and roll rotations, the sheet moves along Z axis with the constant velocity *v*_2_. Length of the shearing line amounts to *l* = 50 mm, rake angle value is set to α = 7°. To reduce the calculation time, knives are considered as rigid bodies meshed with an 8-node Solid164 element type. After exploratory analyses, a decision was made to generate the mapped mesh with various sheet densities in tools-material contact zones. In case of calculations using the SPH method, it was necessary to create two material domains, in which the particles were tied to the FEM portion of domain using tied types of the contact (Figure 9c).

The Johnson-Cook constitutive equation used in this study, allows to determine the dependence of yield stress on plastic deformation, taking into account the damage of the material [47,61,62,63,64]. The model considers the impact of strain rate and temperature on the yield stress values according to the relationship:

(6) $$\sigma_{Y}=\left(A+B\cdot \varepsilon^{n}\right)\left(1+C\cdot ln{\dot{\varepsilon }}^{*}\right)\left[1-{\left(\frac{T-T_{r}}{T_{m}-T_{r}}\right)}^{m}\right]$$

where *A*, *B*, *C*, *n*, and *m* are the Johnson-Cook constitutive model constants, ε is the equivalent plastic strain, ${\dot{\varepsilon }}^{*}$ is the normalized effective plastic strain rate, and *σ_Y_* is the yield stress [47], *T* is the workpiece temperature, *T_m_* is the material melting temperature, *T_r_* is the room temperature. For AA6111-T4 steel: *A* = 324.1 MPa, *B* = 113.8 MPa, *C* = 0.002, *m* = 1.34, *n* = 0.42 [62]. Constant coefficients of static friction *μ_s_* = 0.08 and kinetic friction *μ_d_* = 0.009, were accepted and described using Coulomb’s friction model.

## 4. Numerical Results

**Figure 11:**
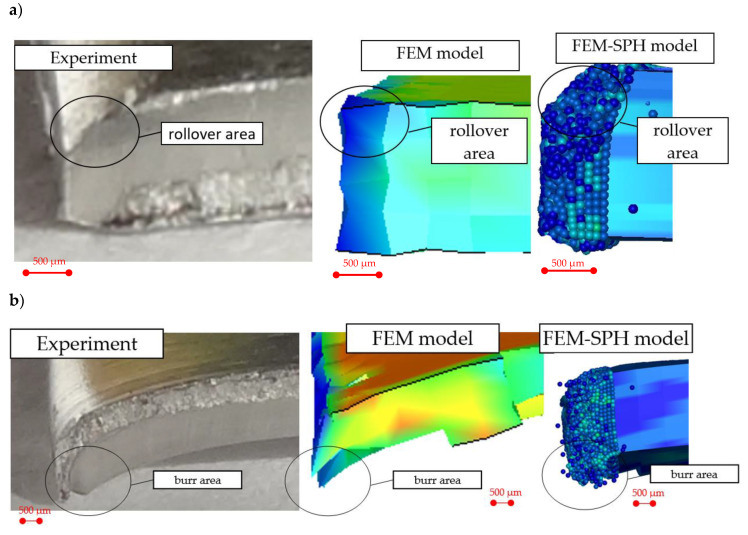
Characteristic features of the sheared edges obtained experimentally and numerically: (**a**) h_c_ = 0.06 mm, (**b**) h_c_ = 0.09 mm.

**Figure 12:**
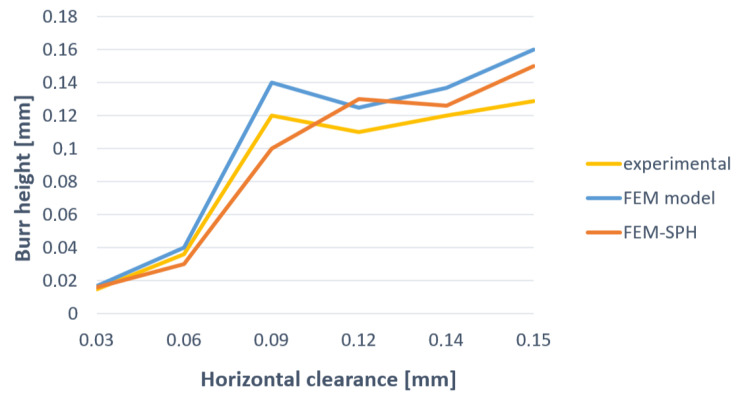
Graph of burr height obtained numerically and experimentally.

The results of numerical simulations are shown in Figure 11 and compared with the ones obtained experimentally. The analysis of the results shows that both the FEM and SPH methods accurately predict the characteristic features of the cut edge. The highest accuracy was obtained in the rollover and burr formations’ mechanisms. From a practical point of view, correct burr prediction is particularly important. The Figure 12 presents validation of the FE and SPH models based on the comparative analysis of the burr height. As indicated in experimental studies, as the clearance increases, the burr height increases, what is reflected in the FEM and SPH models. The comparison between the experimental and predicted by models burr height values shows, that the differences do not exceed 15%.

**Figure 13:**
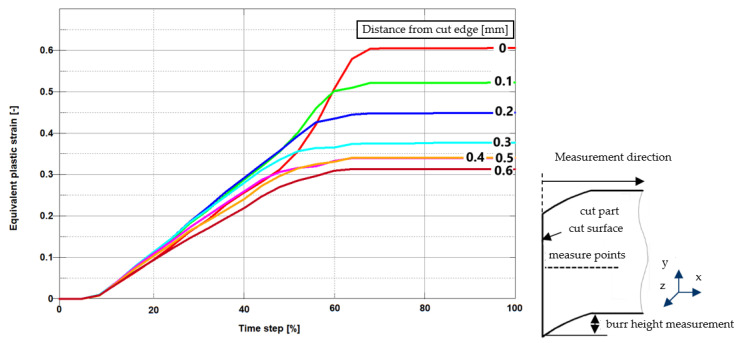
Equivalent plastic strain on sheared edge measured for h_c_ = 0.06 mm (FEM model).

**Figure 14:**
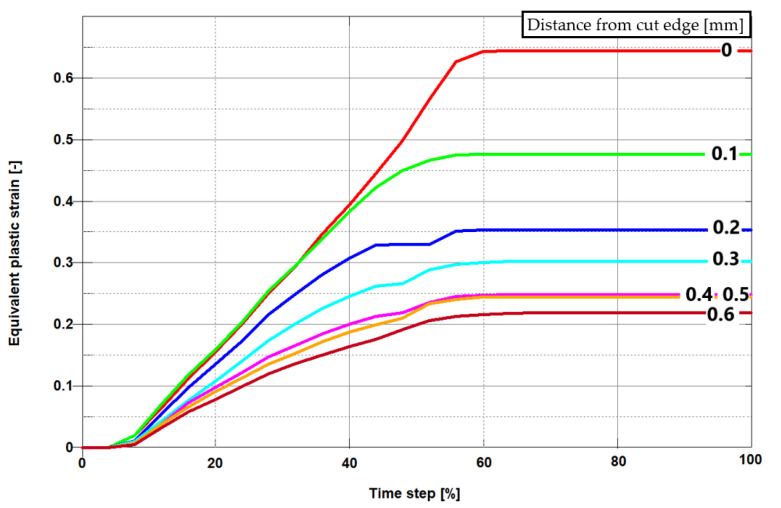
Equivalent plastic strain on sheared edge for h_c_ = 0.06 mm (FEM + SPH model).

In Figure 13 and Figure 14 there are presented the effective plastic strain values obtained from FEM and FEM-SPH models, measured at the points gradually spaced from the cut edge after the process. The maximum effective plastic strain value from both the models amounted approximately to 0.6 and is concentrated directly on the cut edge. It decreases together with the depth of the material. Stabilization occurs approximately at a depth from the cut edge 0.35–0.4 mm in both models. Some of the differences between the strain characteristics obtained from FEM and FEM-SPH models, resulted from friction characteristics, particle density, and SPH viscosity parameters. 

**Figure 15:**
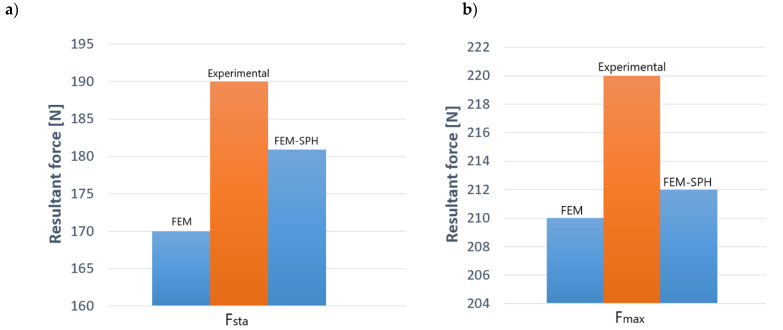
A comparison of the resultant force of the experiment and the simulation: (**a**) cutting force at steady state, (**b**) maximum force.

In order to validate the measured force characteristics of the models, experimental data were compared in all the numerical simulations. In the FEM and FEM-SPH modeling, the resultant forces were analyzed. The experimental verification of the cutting force at steady state is presented in Figure 15a for a selected example. The experimental verification of the maximal cutting force is shown in Figure 15b. Values of forces obtained by numerical calculation show a little difference from the experimental results. The FEM-SPH model predicted these variables more accurately than the FEM model. However, for these models, the difference does not exceed 15%.

## 5. Optimization

**Figure 16:**
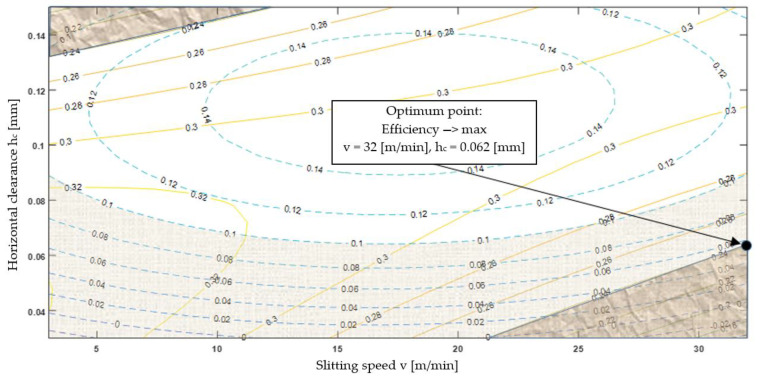
Graphical optimization of the cutting process.

Optimization of the shear-slitting process was carried out on the basis of the developed mathematical models of the process (experimental and numerical). The optimization task is defined as follows: goal function-maximizing the efficiency of the cutting process-efficiency → max. Decision variables are the following: horizontal clearance *h_c_* є [0.03–0.15] mm, and slitting speed *v* є [3–32] m/min. Limitations are the following: burr height *h_b_* < 0.1 mm, width of fracture zone *fr* < 0.24 mm (Figure 16). The task defined in this way makes it possible to reach high workpiece’s technological quality, low costs of tool production, and to obtain high process efficiency. Graphic optimization was used to solve this task [65]. It allows for the easily determination of the area of acceptable solutions and optimal parameters of decision variables.

This solution is particularly useful for production reasons, as it allows to make decisions easily after analyzing the chart. Following the optimization of the cutting process, the optimal point of the process efficiency (for the highest possible cutting speed *v*) was determined, for which the decision variables assume the values: *v* = 32 m/min, *h_c_* = 0.062 mm.

## 6. Conclusions

Despite of the development of cutting technology, there are still many problems to be solved on production lines. This applies, in particular, to the machining of difficult-to-cut materials, which includes the aluminum alloys. The main problem is that for individual cutting methods and the type of shaped alloy, it is necessary to use different process parameters, the correct selection of which causes problems due to a small number of publications related to the topic. This particularly applies to shear-slitting operations. This process is characterized by complex kinematics, due to using the machining parameters optimum for cutting with other techniques, which does not warrant obtaining high-quality products. As a result, in the shearing processes for AA6111-T4 sheets, edge fracture, rollover and burr formations on cut edge are observed. Based on the FEM and SPH methods, physical, mathematical, and numerical models were developed to enable a detailed analysis of these issues. The application of the updated Lagrange description allows for correct formulation of physically and geometrically non-linear dynamics issues. A variational approach to the object’s motion equations is effective, which is the basis for their discretization by the finite element method, and then the solution of the discrete equations has been obtained with the use of the explicit method.

Based on the results of the research, the following conclusions can be formulated:

- The use of two FEM and SPH numerical methods, enabled gaining new knowledge about shear-slitting processes, which can help in choosing the best and the most accurate numerical method to simulate similar processes, especially those ones in which strong deformation, structure fracture and separation occurs.
- The conducted experimental research using vision systems allowed for observing the physical phenomena occurring within very small areas and running at high speeds. Thanks to that, it was possible to learn them more accurately, as well as to use the recorded images to validate simulation models in individual cutting phases. So far, the validation of simulation models has involved comparative analysis of cutting forces and the quality of the cut edge with the experiment offline (after the process). The presented method enables the analysis of accuracy and correctness of simulation models, in unstable phases inclusive, e.g., during separation, online.
- The most important and controllable parameters affecting the quality of the sheared edge include the slitting speed and the horizontal clearance. The results indicate a significant effect of the clearance on the deformation zone’s width. Along with the increase in the clearance value, the deformation zone increased. Conditions affecting burr formation on the edge were also specified. The highest burrs were obtained when the slitting speed was set to *v* = 17 m/min and the clearance was set to *h_c_* = 6%*t*.
- The use of graphic optimization has enabled the determination of process conditions that allow for obtaining the highest quality of the cut edge for the given criteria. The most favorable conditions were obtained for *v* = 32 m/min, *h_c_* = 0.062 mm and *α* = 30°. The proposed methodology can be used to analyze various materials of different thicknesses formed by mechanical cutting.

## Figures and Tables

**Table 1:** Mechanical and thermal properties of AA6111-T4 aluminum alloy (at *T* = 20 °C).

**Young’s Modulus**, *E*	70 GPa
**Elongation**, *A*_100_	28%
**Shear Modulus**, *G*	26–26.5 GPa
**Poisson’s Ratio**, *ν*	0.33
**Tensile Strength**, *R_m_*	265–285 MPa
**Fracture Toughness**, *K_IC_*	22–35 MPa·√m
**Yield Strength Rp0.2**, *Rp*_0.2_	150–170 MPa
**Coefficient of Thermal Expansion**, *α*	1.6E-5–2.4E-5 1/K
**Specific Heat Capacity**, *c_p_*	887–963 J/(kg·K)
**Thermal Conductivity**, *λ*	170–220 W/(m·K)

**Table 2:** The chemical composition of AA6111-T4 aluminum alloy [% weight].

Si	Cu	Zn	Cr	Mn	Ti	Mg	Fe
0.6–1.1	0.5–0.9	0.15	0.1	0.1–0.45	0.1	0.5–1	0.4

**Table 3:** Shear-slitting parameters.

**Horizontal Clearance**, *h_c_*	0.03–0.15 mm
**Vertical Clearance**, *c_v_*	0.15 mm
**Slitting Speed**, *v*_2_	3–32 m/min
**Rake Angle of the Upper Knife**, *α*	5°–40°
**Upper Knife, Polyurethane Roll Radius**, *r*_1_, *r*_2_	15 mm
**Lower Knife Radius**, *r*_3_	20 mm

**Table 4:** Five-level rotatable plan of experiment.

Plan Level	Coded Variables			Real Variables		
	${\overset{⌣}{\bar{x}}}_{1}$	${\overset{⌣}{\bar{x}}}_{2}$	${\overset{⌣}{\bar{x}}}_{3}$	h_c_ [mm]	v_2_ [m/min]	α [°]
1	−	−	−	0.054	8.87	12.09
2	+	−	−	0.125	8.87	12.09
3	−	+	−	0.054	26.12	12.09
4	+	+	−	0.125	26.12	12.09
5	−	−	+	0.054	8.87	32.9
6	+	−	+	0.125	8.87	32.9
7	−	+	+	0.054	26.12	32.9
8	+	+	+	0.125	26.12	32.9
9	+α = 1.682	0	0	0.15	17.5	22.5
10	−α = −1.682	0	0	0.03	17.5	22.5
11	0	+α = 1.682	0	0.09	32	22.5
12	0	−α = −1.682	0	0.09	3	22.5
13	0	0	+α = 1.682	0.09	17.5	40
14	0	0	−α = −1.682	0.09	17.5	5
15	0	0	0	0.09	17.5	22.5
16	0	0	0	0.09	17.5	22.5
17	0	0	0	0.09	17.5	22.5
18	0	0	0	0.09	17.5	22.5
19	0	0	0	0.09	17.5	22.5
20	0	0	0	0.09	17.5	22.5

## References

[1] Legutko S. (2009). Development Trends in Machines Operation Maintenance. Eksploat. i Niezawodn. -Maint. Reliab..

[2] Kumar R., Chatopadhyaya S., Hloch S., Krolczyk G., Legutko S. (2016). Wear characteristics and defects analysis of friction stir welded joint of aluminum alloy 6061-T6. Eksploat. i Niezawodn. -Maint. Reliab..

[3] Maruda R.W., Feldshtein E., Legutko S., Krolczyk G.M. (2015). Research on Emulsion Mist Generation in the Conditions of Minimum Quantity Cooling Lubrication (MQCL). Tehnicki Vjesn. -Tech. Gaz..

[4] Saanouni K. (2006). Virtual metal forming including the ductile damage occurrence. Actual state of the art and main perspectives. J. Mater. Process. Technol..

[5] Bohdal L. (2016). The application of the smoothed particle hydrodynamics (SPH) method to the simulation and analysis of blanking process. Mechanika.

[6] Gontarz S., Gumiński R. (2017). New approach to the evaluation of the effort state of steel based on magneto-mechanical effects. Mech. Res. Comm..

[7] Kukielka L. (2001). Mathematical modelling and numerical simulation of non-linear deformation of the asperity in the burnishing cold rolling operation. Computational Methods in Contact Mechanics V: Proceedings of the 5th International Conference on Computational Methods in Contact Mechanics, Seville, Spain, 7 June 2001.

[8] Aggarwal S.H., Bhushan B., Katsube N. (2005). Three-dimensional finite element analysis of the magnetic tape slitting process. J. Mater. Process. Technol..

[9] Liu C., Lu H., Huang Y. (2005). Dynamic steady-state stress field in a web during slitting. ASME J. App. Mech..

[10] Lu H., Wang B., Iqbal J. Deformation in shear slitting of polymeric webs. Proceedings of the 6th International Conference on Web Handling.

[11] Krinninger M., Steinlehner F., Opritescu D., Golle R., Volk W. (2017). On the influence of different parameters on the characteristic cutting surface when shear cutting aluminum. Procedia CIRP.

[12] Li M. (2000). Micromechanisms of deformation and fracture in shearing aluminum alloy sheet. Int. J. Mech. Sci..

[13] Golovashchenko S.F. (2006). A study on trimming of aluminum autobody sheet and development of a new robust process eliminating burrs and slivers. Int. J. Mech. Sci..

[14] Ma J., Lu H., Li M., Wang B. (2006). Burr height in shear slitting of aluminum webs. ASME J. Man. Sci. Eng..

[15] Wang J.P. (2015). A novel fine-blanking approach. Int. J. Adv. Man. Technol..

[16] Wang N., Golovashchenko S.F. (2016). Mechanism of fracture of aluminum blanks subjected to stretching along the sheared edgel. J. Mater. Proc. Technol..

[17] Mao H., Zhou F., Liu Y., Hua L. (2016). Numerical and experimental investigation of the discontinuous dot indenter in the fine-blanking process. J. Man. Proc..

[18] Shih H.C., Shi M.F. (2011). An innovative shearing process for AHSS edge stretchability improvements. J. Man. Sci. Eng..

[19] Du H., Fan W.F., Zhang Z.M. (2010). Comparative study of the process fracture between fine-blanking with negative clearance and conventional blanking. Adv. Mater. Res..

[20] Le Q.B., de Vries J.A., Golovashchenko S.F., Bonnen J.F. (2014). Analysis of Sheared Edge Formability of Aluminum. J. Mater. Process. Technol..

[21] Nasheralahkami S., Golovashchenko S.F., Pan K., Brown L., Gugnani B. (2016). Characterization of Trimmed Edge of Advanced High Strength Steel.

[22] Golovashchenko S.F. (2008). Quality of trimming and its effect on stretch flanging of automotive panels. J. Mater. Eng. Perform..

[23] Hilditch T.B., Hodgson P.D. (2005). Development of the sheared edge in the trimming of steel and light metal sheet: Part 1—Experimental observations. J. Mater. Proc. Technol..

[24] Hilditch T.B., Hodgson P.D. (2005). Development of the sheared edge in the trimming of steel and light metal sheet: Part 2—Mechanisms and modeling. J. Mater. Proc. Technol..

[25] Kaczmarczyk J., Grajcar A. (2018). Numerical Simulation and Experimental Investigation of Cold-Rolled Steel Cutting. Materials.

[26] Gąsiorek D. (2013). The application of the smoothed particle hydrodynamics (SPH) method and the experimental verification of cutting of sheet metal bundles using a guillotine. J. Theor. Appl. Mech..

[27] Bohdal Ł., Kułakowska A., Patyk R. (2014). Analysis of Slitting of Aluminum Body Panels in the Aspect of Scrap Reduction. Ann. Set Env. Prot..

[28] Bohdal L., Kukielka L. (2015). Application of variational and FEM methods to the modelling and numerical analysis of the slitting process for geometrical and physical nonlinearity. J. Theor. Appl. Mech..

[29] Bohdal L. (2015). Application of FEM and vision-based methods to analysis of shearing processes in the aspect of scrap reduction. Ann. Set Environ. Prot..

[30] Hubert C., Dubar L., Dubar M., Dubois A. (2010). Experimental simulation of strip edge cracking in steel rolling sequences. J. Mater. Proc. Technol..

[31] Górszczyk J., Malicki K., Zych T. (2019). Application of digital image correlation (DIC) method for road material testing. Materials.

[32] Górszczyk J., Malicki K. (2019). Digital Image Correlation Method in monitoring deformation during geogrid testing. Fibres Text. East. Eur..

[33] Malesa M., Malowany K., Kujawińska M. (2014). Multi-camera DIC system with a spatial data stitching procedure for measurements of engineering objects. Photonics Lett. Pol..

[34] Dai S., Liu X., Nawnit K. (2019). Experimental study on the fracture process zone characteristics in concrete utilizing DIC and AE methods. Appl. Sci..

[35] Jang L., Smith L., Gothekar A., Chen X. (2010). Measure Strain Distribution Using Digital Image Correlation (DIC) for Tensile Tests.

[36] Wattrisse B., Chrysochoos A., Muracciole J.M., Nemoz-Gaillard M. (2001). Analysis of strain localization during tensile tests by digital image correlation. Exp. Mech..

[37] Goijaerts A., Govaert L., Baaijens F. (2001). Evaluation of ductile fracture models for diferent metals in blanking. J. Mater. Process. Technol..

[38] Ghadbeigi H., Al-Rubaye A., Robinson F.C.J., Hawezy D., Birosca S., Atallah K. (2020). Blanking induced damage in thin 3.2% silicon steel sheets. Prod. Eng..

[39] Hu X.H., Sun X., Golovashchenko S.F. (2014). Predicted tensile stretchability of trimmed AA6111-T4 sheets. Comp. Mater. Sci..

[40] Yu S., Zhao J. (2012). Investigation on blanking of thick sheet metal using the ductile fracture initiation and propagation criterion. J. Shanghai Jiaotong Univ. (Sci.).

[41] Niu Y., Shao S., Park S.B., Kao C. (2017). A novel speckle-free digital image correlation method for in situ warpage characterization. IEEE Trans. Compon. Packag. Manuf. Technol..

[42] Suliman S.M.A. (2001). An experimental investigation of guillotining of aluminum alloy 5005. Mater. Man. Proc..

[43] Meehan R.R., Burns S.J. (1998). Mechanics of slitting and cutting webs. Exp. Mech..

[44] Wisselink H., Hue’tink J. (2004). 3D FEM simulation of stationary metal forming processes with applications to slitting and rolling. J. Mater. Process. Technol..

[45] Lu H., Ma J., Li M. (2006). Edge trimming of aluminum sheets using shear slitting at a rake angle. ASME J. App. Mech..

[46] Hatanaka N., Yamaguchi K., Takakura N. (2003). Finite element simulation of the shearing mechanism in the blanking of sheet metal. J. Mater. Process. Technol..

[47] Bohdal Ł., Patyk R., Tandecka K., Gontarz S., Jackiewicz D. (2020). Influence of shear-slitting parameters on workpiece formation, cut edge quality and selected magnetic properties for grain-oriented silicon steel. J. Man. Proc. Part A.

[48] Zhao B., Gao G., Gao H., Yan Y. (2014). Finite Element Simulation of Shearing Force in Disc Slitting Process. Adv. Mater. Res..

[49] Zhang M. (2010). Calculation and selection of energetic parameters for disc shears. Heavy Mach..

[50] Ding M., Wu D., Qin Q. (2012). Finite Element Modeling of Shear-Slitting Process. Adv. Mater. Res..

[51] Ghozzi Y., Labergere C., Saanouni K., Parrico A. (2014). Modelling and numerical simulation of thick sheet double slitting process using continuum damage mechanics. Int. J. Dam. Mech..

[52] Bohdal L., Kukiełka L. (2014). Application of variational and FEM methods to the modelling and numerical analysis of guillotining process for geometrical and physical nonlinearity. Mechanika.

[53] Kleiber M. (1985). Finite Element Method in Non-Linear Solid Mechanics.

[54] Kukiełka L., Geleta K., Kukiełka K. (2012). Modelling of initial and boundary problems with geometrical and physical nonlinearity and its application in burnishing processes. Steel Research International, Metal Forming 2012.

[55] Kukiełka L., Kułakowska A., Patyk R. (2010). Numerical Modeling and Simulation of the Movable Contact Tool-Worpiece and Application in Technological Processes. J. Syst. Cyb. Inf..

[56] Kukiełka L. (2010). New damping models of metallic materials and its application in non-linear dynamical cold processes of metal forming. Steel Research International, Proceedings of the 13th International Conference Metal Forming 2010, Toyohashi, Japan, 19–22 September 2010.

[57] Kukiełka L., Geleta K., Kukiełka K. (2012). Modelling and analysis of nonlinear physical phenomena in the burnishing rolling operation with electrical current. Steel Research International, Metal Forming 2012.

[58] Kułakowska A., Kukiełka L., Patyk R. Numerical analysis and experimental researches of burnishing rolling process of workpieces with real surface. Proceedings of the 5th International Symposium on Management, Engineering and Informatics: MEI 2009, The 13th Multi-Conference on Systemics, Cybernetics and Informatics: WMSCI 2009.

[59] Bohdal L., Kukiełka L., Radchenko A.M., Patyk R., Kułakowski M., Chodór R. (2019). Modelling of guillotining process of grain oriented silicon steel using FEM. AIP Conf. Proc..

[60] Bohdal L., Kukiełka L., Świłło S., Radchenko A.M., Kułakowska A. (2019). Modelling and experimental analysis of shear-slitting process of light metal alloys using FEM, SPH and vision-based methods. AIP Conf. Proc..

[61] Johnson G.R., Cook W.H. (1985). Fracture characteristics of three metals subjected to various strains, strain rates, temperatures and pressures. Eng. Fract. Mech..

[62] Bohdal Ł. (2018). Teoretyczne i Doświadczalne Podstawy Optymalizacji Procesów Cięcia Mechanicznego Stopów Metali Lekkich i stali Elektrotechnicznych.

[63] Gasiorek D., Baranowski P., Malachowski J., Mazurkiewicz L., Wiercigroch M. (2018). Modelling of guillotine cutting of multi-layered aluminum sheets. J. Man. Proc..

[64] Bohdal L., Kukielka L., Kukielka K., Kulakowska A., Malag L., Patyk R. (2014). Three Dimensional Finite Element Simulation of Sheet Metal Blanking Process. Appl. Mech. Mater..

[65] Kulakowska A., Patyk R., Bohdal L. (2014). Application of Burnishing Process in Creating Environmental Product. Ann. Set Environ. Prot..

